# A Helpful Measure to Measure Help: The Construction and Validation of the Intergroup Giving and Intergroup Acting in Favor of Refugees Scale (IGIAF)

**DOI:** 10.5334/irsp.832

**Published:** 2024-05-08

**Authors:** Mado Hanioti, Antoine Roblain, Assaad Azzi, Laurent Licata

**Affiliations:** 1 CESCUP, BE; 2 FNRS-FRESH Candidate, BE

**Keywords:** Scale Construction, Intergroup Helping, Intergroup Giving, Intergroup Acting

## Abstract

Since 2015, Europe has experienced two important influxes of vulnerable migrants, refugees, and asylum seekers. Despite heterogenous reactions within and across countries, acts of humanitarianism and solidarity have occurred in a diverse range of behaviors. Given the particularities of Intergroup Helping in favor of refugees, a more nuanced understanding of intentions to engage in helping behaviors would enrich theoretical and applied research. We developed and validated a scale of Intergroup Helping in favor of refugees, that covers the two dimensions of help, i.e., Intergroup Giving (alleviating the suffering of others) and Intergroup Acting (addressing injustices and inequalities). Following scale construction practices, we proceeded in three phases. First, we identified and defined our domains of interest, and proceeded to collect representative helping behaviors, using secondary data of semi-structured interviews on volunteers. These behaviors were pre-tested. Then, across two studies, we examined the scale’s reliability, dimensionality, and validity qualities. Study 1 was distributed among a student sample at two time-points. Results yielded robust internal consistency, test-retest reliability, and predictive validity, and displayed preliminary evidence of the bidimensional structure. Study 2 was distributed among a non-student sample and supported the bidimensional structure of the scale. This research demonstrates that Intergroup Helping intentions in favor of refugees can be classified into Intergroup Giving and Intergroup Acting behaviors and offers a predictive tool to investigate these behaviors in an intergroup context.

## Introduction

Since 2015, the arrivals of migrants, refugees, and asylum seekers increased in an unprecedented manner in Europe. This phenomenon is referred to as the *refugee reception crisis* (Ambrosini et al., 2019; Kang, 2021). Although this is not the first wave of migration Europe has faced, this one stirred heterogeneous attitudes and reactions across and within national boundaries, not only at the institutional level, but also at the individual level (Ambrosini et al., 2019). Individual and collective initiatives surfaced to address short-to-long-term needs of the newcomers because some perceived State inactiveness as unacceptable, as was the case in Belgium (Mescoli & Roblain, 2021).

In Brussels, the amassment of migrants in a ‘migrant camp’ due to limited numbers of authorized applications for asylum seekers led to the emergence of a citizen initiative, the Bel Refugees. This initiative emerged to organize and accommodate these migrants. Other association followed, such as the *CIRE, Médecins Sans Frontières* and *Médecins du Monde*. These initiatives rose because citizens and volunteers perceived that the federal government did not meet the arrivals’ needs and failed to implement necessary policies (Mescoli & Roblain, 2021). Such initiatives offer accommodation, attend to human basic needs, and engage in political and advocacy activities (Mescoli, Roblain & Griffioen, 2020). Similar acts of solidarity occurred across Europe (Kende et al., 2017; Knab & Steffens, 2021).

Such instances of Intergroup Helping offer a possibility to study intergroup dynamics (Van Leeuwen & Zagefka, 2017). Helping contexts not only render salient group memberships, differential statuses, and intergroup relations, but these differences also become a basis for categorization (Kutlaca et al., 2020). Undoubtedly, the refugee reception crisis highlights group memberships and differences between helpers and refugees. Indeed, negative national and individual responses against the acceptance of refugees were based on intergroup issues such as ethnic origin, culture, and religion (Costello & Foster, 2022). It is therefore important to shift from an interpersonal to an intergroup approach to study helping behaviors in favor of refugees (Van Leeuwen & Zagefka, 2017).

In order to stimulate or enhance prosocial attitudes and behaviors towards refugees, it is essential to disentangle underlying drives and motivations. Although one cannot directly affect behavioral outcomes, it is possible to influence peoples intentions to act, and intentions partly lead to actual outcome (Sheeran & Webb, 2016). Indeed, beliefs, attitudes, and affects have a direct impact on people’s intentions, which then can lead to behavioral outcomes (Morwitz & Munz, 2021). Therefore, studying which factors enhance or hinder people’s intention to engage in Intergroup Helping could, theoretically, provide us with ways to boost these behaviors in real life.

This paper contributes to the research in Intergroup Helping by developing and validating a scale that simultaneously considers the two dimensions of Intergroup Helping in favor of refugees. We refer to these dimensions as *Intergroup Giving* and *Intergroup Acting*. This paper reports the construction and validation of the Intergroup Giving and Intergroup Acting in Favor of refugees scale, the *IGIAF*.

## Components of Intergroup Helping

Intergroup helping behaviors are commonly conceptualized as possessing two dimensions. Researchers distinguish benevolence from activism, dependency-oriented from autonomy-oriented behaviors, and hierarchy-maintaining from hierarchy-challenging behaviors (Knab & Steffens, 2021; Nadler, 1998). Benevolence-related and activism-related behaviors are linked to discrete latent profiles, are stimulated by different drives, and lead to distinctive outcomes and consequences (Halabi & Nadler, 2017; Thomas & McGarty, 2018; Van de Vyver & Abrams, 2015). However, both types of behaviors often co-exist (Vandevoordt & Verschraegen, 2019). Benevolent-related and activism-related help complement each other and are equally important.

Benevolence, dependent-oriented or hierarchy-maintaining behaviors refer to the fact that the helpees rely on the helpers for assistance, and result in the fact that the offered help maintains existing hierarchy discrepancies between helpees and helpers (Knab & Steffens, 2021; Nadler, 1998). This type of assistance offers temporary but immediate solutions. When provided with money and clothes, a person is equipped against starvation and the elements, but when the money runs out and the clothes become ragged, that person is yet again dependent on their helpers.

In contrast, activism, autonomy-oriented, and hierarchy-challenging behaviors render the helpees independent and self-reliable and can challenge existing hierarchies by offering lasting—albeit lengthier—solutions (Knab & Steffens, 2021; Nadler, 1998). Protesting for equal rights or citizenship offers better opportunities and protection but does not happen overnight.

Evidence suggests the existence of two distinct latent profiles of helpers, benevolent supporters, and activist supporters (Thomas & McGarty, 2018). Benevolent supporters tend to only engage in benevolent support and hierarchy-maintaining actions, such as giving medical assistance to refugees. These behaviors appear to be specifically predicted by sympathy and empathy. Activist supporters tend to engage in both benevolent and activist support, and thus in hierarchy-maintaining actions, such as helping refugees fight for their rights. Behaviors related to activist support appear to be specifically predicted by moral outrage (Knab & Steffens, 2021; Thomas & McGarty, 2018).

Additionally, evidence suggests that preferences to engage in specific Intergroup Helping behaviors depend on group memberships. Members of high-status groups prefer to offer dependency-oriented help to members of low-status outgroups. In contrast members of minority groups, compared to members of majority groups, seem more prone to engage in collective actions in favor of other minority groups (Halabi & Nadler, 2017; Starzyk et al., 2019).

Finally, research has also shown that, not only do recipients (of intergroup) help react differently to distinct types of helping, but distinct types of helping also have different psychological outcomes for the helpers (Gilster, 2012; Halabi & Nadler, 2017). For instance, members of low-status groups tend to react more positively to autonomy-oriented helping behaviors than to dependency-oriented helping behaviors.

## Current Research and Overview

Typically, research on helping has tended to focus on interpersonal motives and behaviors. It has also tended to separate benevolence-related and activism-related actions (Bales, 1996; Butt et al., 2017; Cervone et al., 2023; Clary et al., 1998; Cnaan & Goldberg-Glen, 1991; Ekman & Amnå, 2012; Talò & Mannarini, 2015; Van Zomeren, Spears & Leach, 2008). It is in the last decades that interest in Intergroup Helping has risen.

Recently, Maki et al. (2017) built an inventory that categorizes people depending on their propensities towards autonomy-oriented, dependency-oriented or opposition towards help, e.g., ‘I help others so that they can learn to solve their own problems,’ ‘I help others because I like solving other people’s problems,’ ‘Helping other people only makes them more needy in the future.’ This inventory investigates interpersonal orientations and how these are linked to helping experiences, or to the maintenance of a helping behavior over time. As shown by the exemplified items, the scale does not measure intentions to engage in (intergroup) helping behaviors. Although Kende et al. (2017) proposed a bidimensional self-developed scale to measure volunteerism and political activism in favor of refugees, this scale was not validated.

It therefore seemed pertinent to develop a scale that considers the nature of helping behaviors, that incorporates both dimensions of helping, i.e., Intergroup Giving and Intergroup Acting, and that is suited to an intergroup approach.

To develop and validate the Intergroup Giving and Intergroup Acting in Favor of refugees scale (IGIAF), we followed a stepwise procedure of best practices and recommendations, as per Boateng and colleagues (2018).

Accordingly, we first focused on the generation and selection of the initial item pool. Second, we administered the scale to a student sample and performed an exploratory factor analysis (EFA). Third, we conducted a confirmatory factor analysis (CFA) on a non-student sample to test the hypothesized bidimensional structure. Finally, additional analyses of reliability (internal consistency and test-retest reliability) and of validity (e.g., criterion and construct validity) were also conducted, as detailed below.

### Phase 1: Domain Identification, Item Generation and Item Pre-Testing

Item development includes the identification of the domain of interest and the generation of items. This can be summarized as the selection of appropriate items related to the domain of interest, and the assessment of these items (Boateng et al., 2018).

The aim of Phase 1 was to first define the domain of interest, then to generate potential items related to the domain of interest, and to pre-test these items while testing for face validity.

#### Defining the Domain of Interest

First, we delineated and defined the domain of interest. Our aim was to offer two dimensions that might better reflect behaviors observed in the field, without limiting behaviors to an apolitical versus political division. Based on the literature review, we outlined two dimensions of Intergroup Helping. The first dimension is *Intergroup Giving:* behaviors akin to volunteerism, charity and philanthropy, aimed at alleviating the suffering of the recipients by addressing basic human needs such as food, shelter, clothing, and psychological support. This definition accommodates fundamental elements that combine previous definitions of benevolence and volunteerism. Namely, the concern for the welfare of the beneficiaries and the aim to alleviate their suffering (Livnat, 2004), by offering one’s time freely (Cnaan et al., 1996).

The second dimension is *Intergroup Acting:* actions in favor of the recipients, aimed at addressing social injustices and inequalities. This designation incorporates elements of prior definitions of activism and collective action, such as, pursuing collective interests, addressing a problem linked to (collective) injustices or disadvantages, and being oriented towards change (Snow & Oliver, 1995).

This distinction does not limit itself to an apolitical and political categorization. The reason for this is that the refugee reception crisis underscored that behaviors that are usually considered humanitarian can become politicized, while some actions that are typically considered political can be conducted with no political motives in mind (Vandevoordt & Verschraegen, 2019). For instance, people volunteered to host refugees to oppose governmental decisions and others demonstrated for purely humanitarian reasons.

#### Item Generation

The second step consisted of generating the item pool. We opted for an inductive method using interviews conducted on the target population (Boateng et al., 2018). This ensures a subtle link between items and construct definitions (Hinkin, 1995). Qualitative techniques offer the possibility to identify manifest forms of the behaviors. This would ensure the use of items that reflects actuals behaviors that occur in Belgium.

We examined semi-structured interviews that had been collected in 2015 by students enrolled in a sociology course at the *Université Libre de Bruxelles*. Participants had been asked semi open-ended questions by the students concerning how they had helped or were helping refugees. The interview guide was structured as such: ‘You are a member of the [names of the two main associations helping refugees active at that time], correct? What exactly do you do there? Could you elaborate more? How long have you been volunteering there? How did you discover the place? Do you volunteer elsewhere?’

The interviewees were people that had helped refugees in 2015, e.g., people who had housed refugees, people who had offered their expertise for free, etc. We collected the list of behaviors they reported engaging in or having engaged in during the past.

The sample consisted of 19 interviews (8 women and 11 men). Theoretical saturation was met at 72 behaviors. Theoretical saturation is obtained when additional data do not provide additional information related to the subject of interest (Strauss & Corbin, 1998). We retained behaviors that had been cited at least three times, leaving a total of 29 items.

#### Pre-Testing the Items

To evaluate face validity, we distributed an online survey where participants were asked to assess each of the 29 items. This aimed to identify which behaviors/items characterized Intergroup Giving and which items characterized Intergroup Acting behaviors. Face validity is the ‘degree that respondents or end users [or lay persons] judge that the items of an assessment instrument are appropriate to the targeted construct and assessment objectives’ (Boateng et al., 2018: 7). We distributed an online survey to students enrolled in a bachelor’s degree in psychology in Belgium^1^ (*n* = 108, *M*_age_ = 21.3, *SD* = 5.09, 94 women, 14 men, 1 other).

Participants received a simplified definition for each dimension: i.e., Intergroup Giving is ‘any humanitarian aid behavior similar to philanthropy or charity;’ Intergroup Acting is ‘any helping behavior with socio-political connotations, ranging from social action to political action.’ For each behavior, participants evaluated the degree to which they considered it to be characteristic of an *Intergroup Giving* behavior, and the degree with which they considered the same behavior to be characteristic of an *Intergroup Acting* behavior.

We conducted Wilcoxon Signed-Ranked tests to keep items that were evaluated significantly differently on these two dimensions. The Wilcoxon Signed-Ranked test is more robust in case of violation of the assumption of normality of distribution (Ayinde, Adejuno & Solomon, 2016). We retained seven items that were evaluated as being characteristic of Intergroup Giving behaviors and seven items that were evaluated as Intergroup Acting behaviors. The list of assessed items can be found in the supplementary materials.

## Study 1: Phase 2, Exploratory Factor Analysis and Assessments of Validity

In Phase 2, as per Boateng et al.’s recommendations (2018), we administered a survey at two different time-points (Study 1). Phase 2 included the exploratory factor analysis (EFA), assessments of the items through inter-item and item-total correlations, assessments of reliability, and validity.

### Method

#### Participants and Procedure

The sample consisted of a total of 330 participants enrolled in a bachelor’s degree in psychology at the *Université Libre de Bruxelles* (278 women, 43 men, 2 non-binary, 1 other, 2 did not disclose). Age ranged from 17 to 33 years old (*M_age_* = 19.30, *SD_age_* = 2.03). Within the sample, 71.5% reported being Belgian, 6.4% from France and the rest reported being from around the world (e.g., Morocco, USA, Pakistan). Participants were administered two online surveys, one month and two weeks apart, in French. At the end of the second survey, they received a written debrief. Two hundred fifty-six participants responded to both time slots, 61 participants responded only to timeslot one and 13 participants responded only to timeslot two.

#### Measures

Unless specified, participants responded with a 7-point scale, ranging from *1 Not at all, 4 Neutral*, to *7 Totally*.

*Intentions to engage in Intergroup Giving behaviors* were measured with the following instruction:

‘We are currently amid a migration crisis, or migratory reception crisis. Since 2015, large numbers of refugees and asylum seekers have been coming to Europe to flee their countries for a variety of reasons. Referring to this migration crisis, please indicate, for each of the following behaviors, the degree to which you intend to engage in this behavior in the near future (within the next 6 months).’

Participants were shown a list of seven behaviors, e.g., ‘Working as a volunteer in an organization (ex: handle the reception desk, take care of logistics, do the cleaning, etc.).’ These were measured at both time-points.

*Intentions to engage in Intergroup Acting behaviors* were measured with the same instruction as for Intergroup Giving. Participants were shown a list of seven behaviors, e.g., ‘Petition to express political discontent (ex: signing a petition for the opening of the borders in the context of the migration crisis, etc.).’ These were measured at both time-points.

*Actual Intergroup Giving behaviors*. Participants were asked the frequency with which they engaged in the seven Intergroup Giving behaviors. The instruction read ‘Please indicate the frequency with which you engaged in the following behaviors in the last 6 months.’ These were measured at the both time-points.

*Actual Intergroup Acting behaviors*. Participants were asked the frequency with which they engaged in the seven Intergroup Acting behaviors. The instruction was the same as for Actual Intergroup Giving behaviors. These were measured at both time-points.

*Six volunteerism* and *six activism* items of Kende et al.’s study (2017) were also measured, e.g., ‘I helped refugees in the field (e.g., train stations, refugee camps, headquarters of civil organizations,’ ‘Did you post or share political contents connected to the refugee crisis on your own Facebook page/Twitter account/blog?’ These were measured at the first time-point to assess convergent validity.

*Self-reported identification* as a volunteer and self-reported identification as an activist were measured at the first time-point, ‘Please indicate the extent to which you agree with the following statements: ‘I identify as a “volunteer,”’ ‘I identify as an “activist.”’ This was to test for differentiation by known groups.

*Self-reported membership*. Participants were asked if they were members of any organization that helps refugees with a single item: ‘Are you a member of an organization that helps refugees? [Yes/No] \.’ These were measured at the first time-point.

*Demographic information* was collected on age, gender, national origins, nationality, level of education and language proficiency.

#### Analytic Strategy

We conducted an EFA to determine the optimal number of factors. We assessed the items to ensure that the selected items were functional, parsimonious, and internally consistent. Then, we tested the reliability of the scale for internal consistency and the stability of intraindividual responses across time. Finally, we assessed validity properties of the scale, namely predictive validity, criterion validity, and three measures of construct validity (Boateng et al., 2018).

### Results

We conducted an EFA and assessed inter-item and item-total correlations for the seven pre-tested items of Intergroup Giving and the seven pre-tested items of Intergroup Acting. We tested the internal consistency of the two sub-scale with MacDonald’s omega. To test for the validity properties of the subscales, we assessed test-retest reliability analyses using Pearson’s correlations, predictive validity of Intergroup Giving and of Intergroup Acting with linear regressions, convergent validity through Pearson’s correlation of the subscales with similar constructs (volunteerism and activism), and differentiation by known groups with linear regressions, as detailed below.

#### Factor Extraction Analysis

We ran an EFA, specifying a Principal Axis extraction, an Oblimin Rotation based on parallel analysis, and a factor loading cut off at >.30 (Crawford et al., 2010; Ledesma & Valero-Mora, 2019).

The EFA extracted two factors, presented in Table 1. Kaiser-Meyer-Olkin (KMO) indexes ranged from .78 to .95. Bartlett’s Test of Sphericity was significant, *χ^2^*_(91)_ = 2135, *p* < .001, confirming that the dataset was suitable for performing an EFA. The inter-factor correlation was significant but not too high, *r* = .51, *p* < .001, supporting the use of a Oblimin rotation^2^ (Brown, 2015; Osborn, 2015; Watson, 2017).

**Table 1:** Exploratory Factor Analysis: Factor Loadings and Communalities of the Dimensions Intergroup Giving and Intergroup Acting without Intergroup Acting Item 1.


		FACTOR 1	FACTOR 2	UNIQUENESS

Intergroup Giving Item 1	Working as a volunteer in an association (e.g., greeting people, cleaning the premises, taking care of logistics, etc.)	.74355	–.0213	.462

Intergroup Giving Item 2	Collecting donations (e.g., going door to door, soliciting people on the street, organizing a charity event, etc.)	.49820	.1802	.630

Intergroup Giving Item 3	Preparing hot meals to be redistributed (e.g., to the homeless, to refugees)	.89704	–.0618	.247

Intergroup Giving Item 4	Donating money, food, clothes, toys, etc.	.37034	.2301	.725

Intergroup Giving Item 5	Participating in the distribution of clothes, meals, etc.	.84131	.0176	.277

Intergroup Giving Item 6	Providing temporary accommodation/housing	.35556	.1294	.811

Intergroup Giving Item 7	Providing moral and/or psychological support (e.g., listening to life stories, talking)	.63676	.0638	.550

Intergroup Acting Item 2	Participating in demonstration to show humanitarian support	–.00886	.8180	.338

Intergroup Acting Item 3	Participating in a demonstration (e.g., to show political dissatisfaction with a refusal by the authorities)	–.03781	.8638	.285

Intergroup Acting Item 4	Encouraging people to mobilize to carry out a collective action	.30301	.5776	.400

Intergroup Acting Item 5	Participating in political actions (e.g., voting at local or national level)	–.00680	.6687	.557

Intergroup Acting Item 6	Petitioning to show political discontent	–.08185	.6135	.667

Intergroup Acting Item 7	Spreading information publicly (e.g., sharing information on social networks, writing an article on the topic, writing in a blog)	.10839	.50028	.683


*Note*. ‘Principal axis factoring’ extraction method was used in combination with a ‘oblimin’ rotation.

All Intergroup Giving items loaded on Factor 1, that explained 26.2% of the variance. Intergroup Acting items 2 to 7 loaded on Factor 2, that explained 22.1% of the variance. Intergroup Acting item 1 loaded on the Factor 1.

We ran a second EFA, excluding Intergroup Acting Item 1. Results yielded a two-factor solution, presented in Table 1. Factor 1 explained 25.1% of the variance, with all Intergroup Giving items loading on this Factor, and Factor 2 explained 23.9% of the variance with all Intergroup Acting loading on this Factor. The KMO measures of sphericity remained significant, *χ^2^*_(78)_ = 1984, *p* < .001, and Inter-Factor correlation became *r* = .50.

#### Test of Reliability

We used McDonald’s omega to determine internal consistency as it is more robust than Cronbach’s alpha against deviations from the assumptions of normality and homogeneity of variances (McNeish, 2018; Yang & Green, 2011).

We conducted two reliability analyses on the data of the first time-point, that yielded good results for internal consistency for both dimensions, Intergroup Giving, McDonald’s *ω* = .85, Intergroup Acting, McDonald’s *ω* = .85. Table 2 and Table 3 report McDonald’s *ω* per item if deleted, inter-item and item-total correlations of the two subscales. As shown, inter-item and item-total correlations were globally moderate to high. Intergroup Acting item 1 presented three low inter-item correlations, presented the lowest item-total correlation, and its deletion enhance the value of McDonald’s *ω*, further justifying its exclusion.

**Table 2:** Inter-Item Correlations Between Items of Intergroup Giving on the Left, and Inter-Item Correlations of Intergroup Acting on the Right.


	1	2	3	4	5	6	7		1	2	3	4	5	6	7

**1.** Int. Giving Item 1	–							**1.** Int. Acting Item 1	–						

**2.** Int. Giving Item 2	.45	–						**2.** Int. Acting Item 2	.31	–					

**3.** Int. Giving Item 3	.64	.49	–					**3.** Int. Acting Item 3	.31	.87	–				

**4.** Int. Giving Item 4	.32	.29	.45	–				**4.** Int. Acting Item 4	.44	.56	.59	–			

**5.** Int. Giving Item 5	.64	.46	.74	.44	–			**5.** Int. Acting Item 5	.19	.41	.42	.45	–		

**6.** Int. Giving Item 6	.33	.32	.32	.20	.34	–		**6.** Int. Acting Item 6	.25	.45	.49	.49	.45	–	

**7.** Int. Giving Item 7	.47	.42	.57	.32	.56	.34	–	**7.** Int. Acting Item 7	.27	.36	.39	.39	.31	.56	–


*Note*. Int. Giving, Intergroup Giving; Int. Acting, Intergroup Acting.

**Table 3:** Inter-Total Correlations Between Items of Intergroup Giving, on the Left and Inter-Total Correlations of Intergroup Acting, on the Right.


	MEAN	ITEM-REST CORRELATION	IF ITEM DROPPED		MEAN	ITEM-REST CORRELATION	IF ITEM DROPPED
	
			MCDONALD’S ω				MCDONALD’S ω

Int. Giving Item 1	4.11	0.670	0.821	Int. Acting Item 1	3.31	0.387	0.853

Int. Giving Item 2	3.41	0.555	0.839	Int. Acting Item 2	4.71	0.703	0.808

Int. Giving Item 3	4.55	0.766	0.803	Int. Acting Item 3	4.51	0.729	0.803

Int. Giving Item 4	5.75	0.453	0.850	Int. Acting Item 4	4.42	0.702	0.812

Int. Giving Item 5	4.66	0.759	0.804	Int. Acting Item 5	4.86	0.505	0.839

Int. Giving Item 6	2.08	0.412	0.855	Int. Acting Item 6	5.68	0.621	0.825

Int. Giving Item 7	5.26	0.622	0.829	Int. Acting Item 7	5.37	0.522	0.837


*Note*. Int. Giving, Intergroup Giving; Int. Acting, Intergroup Acting.

We conducted test-retest reliability analyses using Pearson’s correlations. Means of Intergroup Giving at the first time-point and at the second time-point were strongly and positively correlated, *r* = .76, *p* < .001. Means of Intergroup Acting at the first time-point and at the second time-point were also strongly and positively correlated, *r* = .73, *p* < .001.

#### Tests of Criterion and Construct Validity

We conducted linear regressions to test for predictive validity, to see if intentions to engage in Intergroup Helping behaviors of the first time-points predicted actual behavioral outcome in the second time-points.

Results indicated a significant effect of intentions to engage in Intergroup Giving at the first time-point on actual engagement in Intergroup Giving at the second time-point, *F*_(1,253)_ = 38.33, *β* = .42, *p* <.001. Intentions to engage in Intergroup Acting at the first time-point did not predict frequency of engagement in Intergroup Giving at the second time-point, *F*_(1,253)_ = .09, *β* = .02, *p* = .761. These results indicate that intentions to engage in Intergroup Giving alone predict actual future engagement in this behavior.

Likewise, intentions to engage in Intergroup Acting at the first time-point significantly predicted engagement in Intergroup Acting at the second time-point *F*_(1,253)_ = 182.09, *β* = .69, *p* < .001, while intentions to engage in Intergroup Giving did not predict behavioral outcome of Intergroup Acting *F*_(1,253)_ = 2.99, *β* = .08, *p* = .131. These results indicate that intentions to engage in Intergroup Acting alone predict future behavioral outcomes in this behavior.

We then assessed convergent validity. We conducted Pearson correlations to test if our measure of Intergroup Giving was significantly correlated with Kende et al.’s (2017) items of volunteerism and to test if Intergroup Acting was significantly correlated with their activism items. Convergent validity is confirmed if the new scale correlates highly with variables that measure the same construct (Raykov & Marcoulides, 2011). Our results indicated that intention to engage in Intergroup Giving (data from the first time-point) and volunteer items of Kende et al. (2017) were highly and positively correlated, *r* = .56, *p* < .001. Intentions to engage in Intergroup Acting (data from the first time-point) and activism items of Kende et al. (2017) were also highly and positively correlated, *r* = .72, *p* < .001.

Finally, we used self-reported identification measures as a *volunteer* and as an *activist* to verify differentiation by known groups. A multiple linear regression revealed that only Intergroup Giving predicted participants’ self-identification as a volunteer, *F*_(1,314)_ = 41.20, *p* < .001; *β* = .40. Intergroup Acting did not predict self-identification as a volunteer, *F*_(1,314)_ = .08, *p* = .778; *β* = –.02.

Likewise, Intergroup Acting alone predicted participants’ self-identification as an activist, *F*_(1,314)_ = 28.75, *p* < .001; *β* = .33, *p* < .001. Intergroup Giving did not predict self-identification as an activist, *F*_(1,314)_ = 3.44, *p* = .065; *β* = .11, *p* = .065.

## Study 2: Phase 3, Confirmatory Factor Analysis and Construct Validity

In Phase 3 we conducted Study 2 with a non-student sample. We assessed the dimensionality of the scale as well as an additional measure of validity.

Confirmatory Factor Analyses (CFA) assesses the dimensionality of a scale by validating the hypothesized structure. We tested our bidimensional structure and verified the pertinence of items on their respective factors.

Discriminant validity tests whether a given concept is different from other concepts (Boateng et al., 2018). We verified if our scale was significantly different from Maki et al.’s (2017) measure, a validated inventory of people’s disposition towards and against helping. We also calculated the Heterotrait-Monotrait ratio of correlations (HTMT) as an indicator of good discriminant validity (Henseler, Ringle & Sarstedt, 2015).

### Method

#### Participants and Procedure

The sample consisted of a total of 370 participants recruited online on the *Foule Factory* platform (194 women, 176 men, 1 other). Age ranged from 18 to 81 (*M_age_* = 42.8, *SD_age_* = 12.9). Except for one American and one Australian, the participants were European, with most participants being of French origin (*n* = 323). All political orientations from extreme left- to extreme right- wing were represented, with left being the highest (*n* = 89). 82.7% of the sample was either currently employed or was retired. The participants were mostly educated, with only 5.7% not having completed high school. After completing the online survey, participants received a written debrief.

#### Measures

Unless specified, participants responded with a 7-point scale, ranging from *1 Not at all, 4 Neutral*, to *7 Totally*.

*Intentions to engage in Intergroup Giving* and in *Intergroup Acting* were measured with the same items as per Study 1.

*The helping orientation inventory* by Maki et al. (2017) was also included in the survey. It measures individuals’ dispositions towards autonomy-oriented help (e.g., ‘Helping others makes them better able to solve their own problems’), dependency-oriented help (e.g., ‘Helping is all about fixing people’s problems for them’), and opposition towards help (e.g., ‘Helping others makes them less able to solve their own problems’). This was to measure divergent validity.

*Attention checks* were placed at the beginning and towards the end of the survey. If both attention checks were failed, the participant was excluded. Six participants were excluded.

*Demographic Information* was collected on age, gender, nationality, second country of origin, level of education, political orientation, field of employment, current employment status, and past engagement in helping refugees.

#### Analytic Strategy

We conducted a CFA to test the structure of the scale that was obtained in Study 1. We then tested the divergent validity of the scale by comparing it with a known measurement, and by calculating the index of HTMT ratio of correlations (Boateng et al., 2018; Henseler, Ringle & Sarstedt, 2015).

### Results

First, we conducted a CFA to test the dimensionality of the scale and test if it supported the hypothesized bidimensional structure. We then compared those results to a unidimensional structure. Second, we assessed divergent validity, by correlating our dimensions with the three dimensions of the HOI (Maki et al., 2017), dispositions towards dependency-oriented help, dispositions towards autonomy-oriented help and opposition towards helping. Finally, we calculated the HTMT ratio of correlations (Henseler, Ringle & Sarstedt, 2015).

#### Confirmatory Factor Analysis

**Figure 1:**
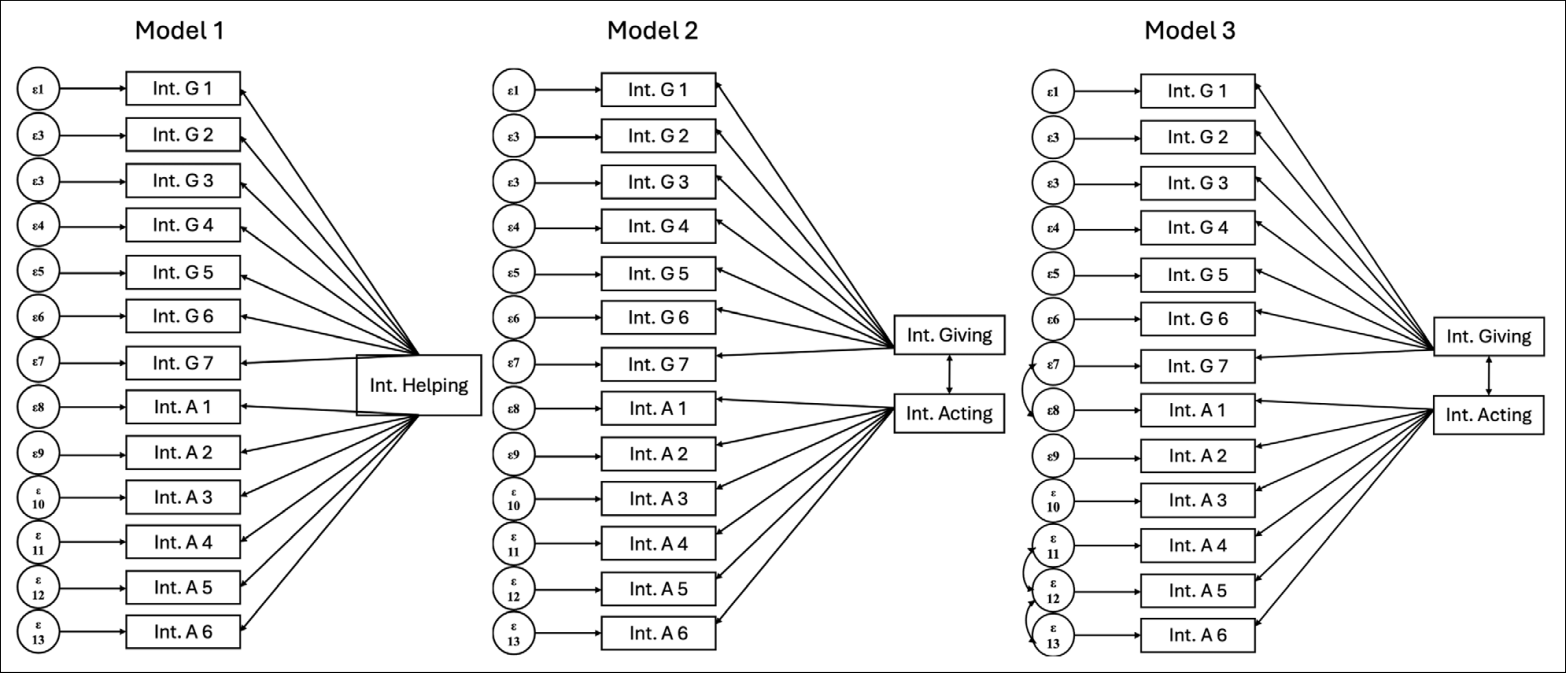
Competing Models of Intergroup Helping. *Note*. IGH, Intergroup Helping intentions; InG, Intergroup Giving; InA, Intergroup Acting; IG 1–7 Intergroup Giving items 1–7; IA 1–6, Intergroup Acting items 1–6. Model 1, one factor; Model 2, two factors, no residual cov.; Model 3, two factor, residual cov. A1–A2, A4–A5, A5–A6.

We conducted CFAs to statistically compare the model fit of three factor configurations (Figure 1) and compared their model fit indices (Table 3).

In the first model (Model 1), all items were forced to load on one factor that represented Intergroup Helping intentions. This yielded a poor fit. In the second model (Model 2), we specified two factors, one for Intergroup Giving items and one for Intergroup Acting items. This configuration represents the theoretical model, and the model suggested by the EFA. Results indicated a moderate fit. All standardized estimates of the indicators were significant at *p* < .001, confirming that each indicator significantly contributed to its respective factor. The factor covariance was significant, *Estimate_Stand_* = .80, *p* < .001.

However, modification Indices of the Residual Covariances suggested to include residual covariances between items IntA1 and IntA2, IntA4 and IntA5, and Int5 and Int6, covariances that all made theoretical sense. Indeed, Item IA 1, *Participating in an event to show humanitarian support*, and Item IA 2, *Participating in a demonstration (to show political dissatisfaction*, are similar behaviors that are driven by different motives. Likewise, Item IA 4, *Participating in political actions*, and Item IA 5, *Petitioning to show political discontent* are both motivated by political drives, and Item IA5 and Item IA 6, *Spreading information publicly*, are both related to opinions.

**Figure 2:**
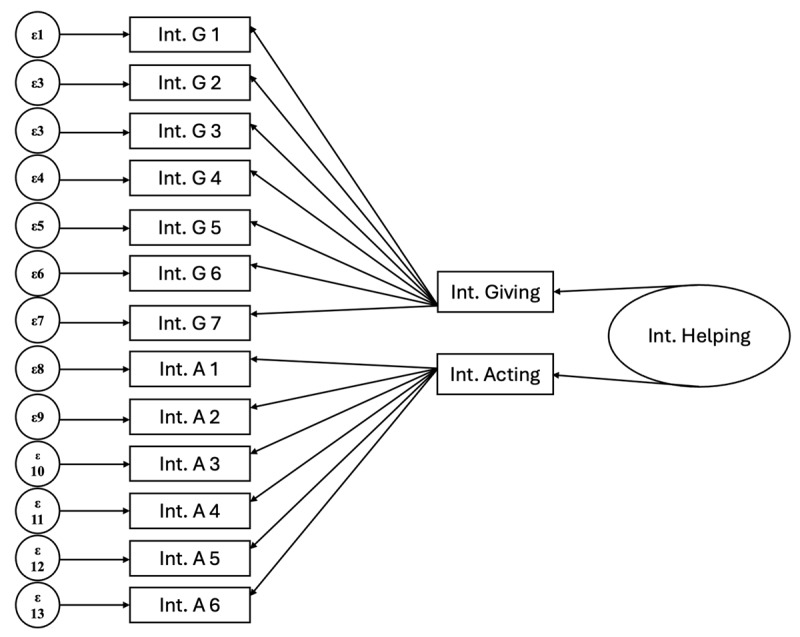
Second Order Model. *Note*. InG, Intergroup Giving; InA, Intergroup Acting; IG 1–7 Intergroup Giving items 1–7; IA 1–6, Intergroup Acting items 1–6.

In the third model (Model 3), we ran the same configuration as Model 2, while permitting covariances of IntA1–IntA2, IntA4–IntA5, IntA5–IntA6. Fit indices improved. We then tested a second-order model (Figure 2) which provided a lower fit than Model 3. This suggests that the scale is best used when measuring Intergroup Giving and Intergroup Acting separately, rather than combining them together as a single measure.

#### Divergent Validity

We conducted Pearson product-moment correlations to assess divergent validity. Discriminant validity is supported when correlations are significant but not too high, ideally <.50–.60 (Akoglu, 2018; Churchill, 1979).

We used Maki et al.’s Helping Orientation Inventory (2017) as the comparative measurement. This inventory measures individual’s dispositions towards dependency-oriented help, autonomy-oriented help and opposition towards helping. We expected intentions to engage in Intergroup Giving to be significantly correlated with dependency and autonomy-oriented disposition, and more highly correlated with dependency-oriented help than when autonomy-oriented help. We expected the opposite for intentions to engage in Intergroup Acting. We also expected Intergroup Giving and Intergroup Acting to be negatively correlated with opposition towards helping. Results of the analyses are found in Tables 4, 5 and 6. The results show that all the correlations were of expected directions and of expected magnitude, confirming divergent validity.

**Table 4:** Competing Models of Intergroup Helping.


FACTOR	INDICATOR	β	SE	LCI	UCI	z	p

***Model 1***							

Intergroup Helping	Int. Giving 1	.76	.08	1.13	1.43	16.90	<.001

	Int. Giving 2	.65	.07	.83	1.10	13.82	<.001

	Int. Giving 3	.83	.08	1.31	1.61	19.28	<.001

	Int. Giving 4	.65	.09	1.07	1.43	13.70	<.001

	Int. Giving 5	.84	.08	1.40	1.71	19.47	<.001

	Int. Giving 6	.64	.07	.80	1.07	13.56	<.001

	Int. Giving 7	.76	.09	1.29	1.62	17.02	<.001

	Int. Acting 1	.84	.08	1.36	1.66	19.50	<.001

	Int. Acting 2	.81	.08	1.33	1.64	18.56	<.001

	Int. Acting 3	.85	.07	1.29	1.57	20.06	<.001

	Int. Acting 4	.57	.11	1.04	1.47	11.61	<.001

	Int. Acting 5	.60	.10	1.06	1.45	12.53	<.001

	Int. Acting 6	.68	.10	1.22	1.59	14.73	<.001

***Model 2***							

Intergroup Giving	Int. Giving 1	.33	.08	.78	1.09	11.93	<.001

	Int. Giving 2	.56	.09	1.42	1.42	12.97	<.001

	Int. Giving 3	.17	.05	.62	.62	9.37	<.001

	Int. Giving 4	.61	.18	2.65	2.65	13.07	<.001

	Int. Giving 5	.17	.06	.70	.70	9.52	<.001

	Int. Giving 6	.63	.10	1.52	1.52	13.10	<.001

	Int. Giving 7	.41	.12	1.76	1.76	12.36	<.001

Intergroup Acting	Int. Acting 1	.19	.07	.48	.74	9.30	<.001

	Int. Acting 2	.20	.07	.53	.80	9.58	<.001

	Int. Acting 3	.25	.07	.58	.85	1.63	<.001

	Int. Acting 4	.65	.24	2.71	3.67	13.09	<.001

	Int. Acting 5	.59	.20	2.18	2.96	12.92	<.001

	Int. Acting 6	.51	.17	1.83	2.50	12.57	<.001

***Model 3***							

Intergroup Giving	Int. Giving 1	.82	.07	1.24	1.52	18.98	<.001

	Int. Giving 2	.66	.07	.84	1.11	14.09	<.001

	Int. Giving 3	.91	.07	1.46	1.74	22.65	<.001

	Int. Giving 4	.62	.09	1.03	1.39	13.03	<.001

	Int. Giving 5	.91	.07	1.55	1.84	22.59	<.001

	Int. Giving 6	.61	.07	.75	1.02	12.70	<.001

	Int. Giving 7	.76	.09	1.30	1.63	17.14	<.001

Intergroup Acting	Int. Acting 1	.85	.08	1.38	1.69	19.81	<.001

	Int. Acting 2	.84	.08	1.39	1.70	19.50	<.001

	Int. Acting 3	.90	.07	1.38	1.65	21.70	<.001

	Int. Acting 4	.58	.11	1.06	1.49	11.66	<.001

	Int. Acting 5	.61	10	1.06	1.45	12.46	<.001

	Int. Acting 6	.71	.10	1.27	1.64	15.20	<.001


**Table 5:** Model Fit of Competing Models of Intergroup Helping Intentions.


	MODEL 1	MODEL 2	MODEL 3	SECOND ORDER MODEL

*X* ^2^	720.09	379.79	241.44	379.79

df	65	64	61	63

*X*^2^/df	11.08	5.93	3.96	6.03

RMSEA	.16	.12	.09	.12

CFI	.81	.91	.95	.91

TLI	.78	.89	.93	.89

SRMR	.07	.06	.95	.06


*Note*. RMSEA, root mean square error of approximation; CFI, comparative fit index, TLI, Tucker-Lewis index; SRMR, Standardized Root Mean Squared.

**Table 6:** Correlations of the Variables Intergroup Giving, Intergroup Acting, Dependency Oriented, Autonomy Oriented, Opposition towards help.


	1	2	3	4	5

**1.** Int. Giving	–				

**2.** Int. Acting	.75	–			

**3.** Dep. Oriented	.38	.29	–		

**4.** Aut. Oriented	.33	.38	.43	–	

**5.** Opposition	–.22	–.33	–.30	–.01^	–


*Note*. Int. Giving, Intergroup Giving; Int. Acting, Intergroup Acting; Dep. Oriented, Dependency Oriented; Aut. Oriented, Autonomy Oriented; Opposition, Opposition towards help; ^non-significant.

As expected, Intergroup Giving was positively correlated with disposition towards Dependency-Oriented help, and this correlation was stronger than with Autonomy-Oriented help. Also as expected, Intergroup Acting was positively correlated with Autonomy-Oriented help, and this was stronger than with Dependency-Oriented help. Finally, both Intergroup Giving and Intergroup Acting were negatively correlated with Opposition towards help.

We calculated the HTMT index of the scale items (Henseler et al., 2015). The results yielded a satisfactory index, HTMT = .721, suggesting good discriminant validity.

## General Discussion

With the increasing and ongoing arrival of refugees, accurately measuring Intergroup Helping is of utmost importance. Yet, to date, no validated measure simultaneously considers the two widely studied dimensions of helping. The present research addresses this issue by introducing and validating a scale to concurrently measure intentions to engage in Intergroup Giving and in Intergroup Acting behaviors in favor of refugees. Following Boateng et al.’s (2018) recommendations and rooting our research in theoretical and empirical literature in social psychology, we developed the scale in three phases. Our results corroborate the pertinence of a bidimensional structure and support the validity of the scale. Thus, we offer a valid measure to assess Intergroup Helping intentions in favor of refugees.

First, we defined our domain of interest. Then, we gathered potential items using secondary data retrieved from the target population, people that had helped refugees during the refugee reception crisis in 2015. This ensured the selection of representative behaviors that reflected concrete forms of engagement in favor of refugees. Then, we pre-tested and evaluated these items to guarantee that the behaviors were relevant to their domain.

Second, we conducted Study 1 that collected data from a student sample at two time-points. This study supported the reliability and bidimensional structure of the scale, and that the scale possessed good predictive, criterion and construct validity properties. Indeed, estimates related to internal consistency were high, and our results yielded good test-retest reliabilities across the two timepoints. The EFA depicted that the items loaded on their respective factors. Intentions at the first time-point predicted actual behaviors at the second time-point, and correctly predicted participants self-identifications.

Third, we carried out Study 2 to conduct a CFA on a non-student sample. The analyses yielded a good fit and indicated that it seems more pertinent to consider a bidimensional construct when measuring Intergroup Helping intentions in favor of refugees than a unidimensional construct. Furthermore, the scale tested well for divergent validity when compared with Maki et al.’s HOI (2017). As the scale was validated in French, it would be cautious to pre-test it if one chooses to use it in other languages. Item bias can be checked, for instance, through linguistic and psychological analyses, differential item functioning analyses, or error analyses (Van de Vijver & Tanzer, 2004).

One limitation of our research is that our scale was tested mainly on educated participants. This leaves the question of its applicability to other populations open to empirical investigation. However, our sample seems representative of the European population to this respect, as 41.2% of Europeans had a tertiary level of education in 2021, of the American population, as 48.4% of Americans had tertiary education or an associate degree in 2021, and of the Canadian population, as 63% had tertiary education in 2022 (Census, 2022^3^; Eurostat, 2022^4^; Statistics Canada, 2023^5^). Although postsecondary education does play a part in voluntarism, money donations, and voting behaviors, its impact is strongest for voting behaviors, and only very modest for voluntarism and donations, with its effect appearing to fade over time (Doyle & Skinner, 2017; Sax, 2004). Furthermore, when considering other factors, variables such as education, age, gender, cultural beliefs, and political beliefs do not significantly impact helping behaviors in favor of refugees (Kossowska et al., 2023).

Another limitation is the addition of the three error-covariances in our model. However, including such covariances, if restricted to when they are theoretically justified, can make sense in some cases, as measures can correlate for reasons that are not primary to the purpose of a scale (Cole, Ciesla & Steiger, 2007). As items IA1 and IA2 both concern types of protesting, items IA4 and IA5 both concern types of actions, and items IA5 and IA6 both include the word ‘political,’ it is likely that they have similar errors in their measurements. Allowing them to covariate not only improved fit indices but probably also provides a more realistic assessment of our model (Bostic, McGartland Rubio & Hood, 2000). Finally, we conducted scale development in the context of Intergroup Helping in favor of refugees, but there is no theoretical reason why it should not translate to broader Intergroup Helping contexts by simple rephrasing of the items, such as inter-ethnic contexts, other minority contexts, or status-related contexts.

Although Intergroup Giving and Intergroup Acting behaviors are correlated, we contend that this does not hinder their theoretical distinction nor their practical usefulness. Indeed, these behaviors often co-occur. For instance, at the individual level, people profiled as ‘activists’ (Thomas & McGarty, 208) tend to engage in both Intergroup Giving and in Intergroup Acting. At the national level, instances of both types of Intergroup Helping are observed at the same time, even if conducted by different actors (Vandevoordt, 2019).

That being said, being able to distinguish between these two dimensions can be very relevant in certain research contexts and is in line with empirical evidence. Indeed, Intergroup Giving and Intergroup Acting are associated with different latent profiles, are motivated by different drives, differ depending on group memberships, and lead to different consequences, as we discussed in the literature review (Halabi & Nalder, 2017; Knab & Steffens, 2021; Starzyk et al., 2019; Thomas & McGarty, 2018).

Correctly studying Intergroup Helping can not only benefit research, but also other actors. For instance, campaigns that raise awareness could better tailor the messages they are trying to convey in accordance with their goals—humanitarian or activist—and to the population they are targeting. If there is an immediate crisis, Intergroup Giving might be the primary goal to achieve. Intergroup Acting can be a path towards social change to address injustices and inequalities. Furthermore, understanding the different consequences of specific types of Intergroup Helping can ensure that the help offered reflects the recipients’ needs and demands to preserve or improve intergroups relations (Halabi & Nadler, 2017).

Understanding how to promote such behaviors can not only have direct implications on refugees’ wellbeing but can also benefit host countries. Engaging in Intergroup Helping behaviors such as Intergroup Giving provides refugees with physical comfort, as well as psychological support, which can not only facilitate their daily lives but also limit their psychological distress (Anjum, Aziz & Hamid, 2023; Shishehgar et al., 2017). Additionally, positive contact with refugees, which can be obtained, inter alia, by volunteering, enhances positive attitudes, solidarity and intergroup emotions towards refugees (Kotzur, Schäfer & Wagner, 2019). Engaging in Intergroup Acting can facilitate refugees’ integration in their host society, with rights giving them access to the public sphere, to social inclusion and to employment which benefit both the refugees and the host countries (Morrice, 2007). Indeed, shortening the waiting time for refugees to integrate the labor market, for instance, can actually reduce the host countries’ welfare expenditures, and can contribute to fill labor shortages (Marbach, Hainmueller & Hangartner, 2018). Although there is an initial cost of accepting refugees in a host country this cost is vastly outweighed by the long term benefits in terms of economic boost, social and cultural diversity.

## Conclusion

In a globalized world where migration patterns never cease to increase, it is important to encourage smooth and positive intergroup dynamics. One positive outcome that research has lately focused on is Intergroup Helping. This paper details the development and validation of a scale to measure Intergroup Helping in favor of refugees. In line with scale recommendations and across two studies on a student and on a non-student sample, our results support the scale properties and its bidimensional structure. The scale simultaneously considers intentions to engage in the two dimensions of Intergroup Helping, referred to as Intergroup Giving and Intergroup Acting behaviors. Finally, as the behaviors observed in Belgium are similar to those observed in the USA by Maki and colleagues (2017) and with those observed in Hungary by Kende and colleagues (2017), we can assume they be generalized to other western countries. Thus, the present research contributes to the field of Intergroup Helping by offering a ‘helpful measure’ to measure help.

## Data Accessibility Statement

Data, materials and scripts are available at: https://osf.io/j2zxd/?view_only=72edb21ec77048139ad9ab503d529d2a.

## Additional File

The additional file for this article can be found as follows:

10.5334/irsp.832.s1 Supplementary Material. Complete List of the 29 Pretested Items, Translated From French for this Paper and Tables A–B.

## Footnotes

The refugee reception crisis was well covered by the Belgian media, with all Belgian residence being aware of the crisis and its repercussions.

We opted for an Oblimin rotation in case the factors were potentially correlated, as is often the case in social studies (Osborn, 2015). If factors are not correlated, an oblique rotation would still be able to assume a correlation of zero and offer a solution similar to an orthogonal rotation (Brown, 2015; Osborn, 2015).

https://www.census.gov/newsroom/press-releases/2022/educational-attainment.html#:~:textn%202021%2C%20the%20highest%20level,college%20but%20not%20a%20degree.

https://ec.europa.eu/eurostat/web/products-eurostat-news/-/ddn-20221114-1.

https://www150.statcan.gc.ca/t1/tbl1/en/tv.action?pid=3710013001.

## Appendix

### Appendix A. The IGIAF scale, translated in English, final version

Instruction: We are currently amid a migration crisis, or migrant reception crisis. Since 2015, many refugees have been coming to Europe to flee their countries for various reasons. Referring to this migration crisis, please indicate, for each of the following behaviors, the degree to which you intend to engage in that behavior in the near future (within the next 6 months).

Item IG 1: Working as a volunteer in an association (e.g., greeting people, cleaning the premises, taking care of logistics, etc.)
Item IG 2: Collecting donations (e.g., door-to-door, soliciting people on the street, organizing a charity event, etc.)
Item IG 3: Preparing hot meals to be redistributed (e.g., to the homeless, to refugees)
Item IG 4: Donating money, food, clothes, toys, etc.
Item IG 5: Participating in the distribution of clothes, meals, etc.
Item IG 6: Providing temporary accommodation/housing
Item IG 7: Providing moral and/or psychological support (e.g., listening to life stories, talking)
Item IA 1: Participating in a demonstration to show humanitarian support
Item IA 2: Participating in a demonstration (e.g., to show political dissatisfaction with a refusal by the authorities)
Item IA 3: Encouraging people to mobilize to carry out a collective action
Item IA 4: Participating in political actions (e.g., voting at the local or national level)
Item IA 5: Petitioning to show political discontent
Item IA 6: Spreading information publicly (e.g., sharing information on social networks, writing an article on the topic, writing in a blog)
