# Supplementary material for: A Helpful Measure to Measure Help: The Construction and Validation of the Intergroup Giving and Intergroup Acting in Favor of Refugees Scale (IGIAF)
Source: Int Rev Soc Psychol. 2024 May 8;37:8. doi: 10.5334/irsp.832 (PMC12372790; doi:10.5334/irsp.832)
Supplement: Supplementary Material. — Complete List of the 29 Pretested Items, Translated From French for this Paper and Tables A–B. [file irsp-37-832-s1.pdf]

Supplementary Material

1. **Complete List of the 29 Pretested Items, Translated From French for this Paper**

You will see items related to helping behaviors. With your cursor, please indicate where you place the item on the different scales ranging from 1 (not at all) to 5 (very much so). Reminder: ‘Giving’ behaviors are akin to humanitarian aid, philanthropy or charity. ‘Doing’ behaviors are akin to socio-political aid. Note: The examples are used to illustrate the item. They are not exhaustive.

[ITEM]

I consider this item to have the characteristics of a Giving behavior:

I consider this item to have the characteristics of a Doing behavior:

1.Accompanying a person in his or her integration, asylum application, social reintegration process (e.g., informing the person of the legal procedures to follow, informing the person of the documentation needed to make a file at the commune, etc.).

2.Accompanying a person physically (e.g., driving the person to medical appointments, accompanying the person to the commune, accompanying the person to training courses, etc.)

3.Spreading information privately (e.g., discussing the migration crisis with family, friends or colleagues)

4. Spreading information publicly (e.g., sharing information on social networks, writing an article on the topic, writing in a blog)

5. Working as a volunteer in an association (e.g., greeting people, cleaning the premises, taking care of logistics, etc.)

6.Collecting donations (e.g., going door to door, soliciting people on the street, organizing a charity event, etc.)

7.Preparing hot meals to be redistributed (e.g., to the homeless, to refugees)

8.Doing cultural promotion (e.g., organize a photo exhibition, organize a conference-debate, create an art project about migrants, etc.)

9.Participating in an event to show humanitarian support (e.g., participate in a march to show support for refugees in the context of the migration crisis)

10.Participating in a demonstration to show political discontent (e.g., participating in a march to open borders in the context of the migration crisis)

11.Participating in the redistribution (e.g., of clothing, hot meals)

12.Participating in sorting (e.g., clothes, food)

13.Making donations (e.g., of money, food, clothing, toys, utensils, materials, blankets, etc.)

14.Entertaining (e.g., organize a concert, a play, a movie, to entertain people in a home)

15.Organizing an activity (e.g., organize a soccer game, a drawing workshop, or any other participatory activity)

16.Housing/hosting (e.g., temporarily hosting a refugee)

17.Informing (e.g., informing refugees of the functioning of an association, of their rights, of the functioning of the STIB)

18.Helping with the installation (e.g., helping to set up tents, toilets, helping to refurbish an apartment that will be used as a place to stay, helping to set up a medical care stand)

19.Offering expertise free of charge (e.g., medical care, legal assistance, translation, psychological consultation, counseling, etc.)

20.Mobilizing people for a collective action

21.Providing moral and/or psychological support (e.g., listening to life stories, talking to refugees at a citizen platform, talking to elderly people in a home, etc.)

22. Political actions (e.g., at local or national level, going on strike)

23.Signing a petition to show humanitarian support (e.g., to show support for refugees)

24.Signing a petition to show political discontent (e.g., signing a petition for open borders in the context of the migration crisis)

25.Being a 'godfather/godmother' (e.g., creating a link, giving advice, listening to the person concerned)

26.Sponsoring (providing financial assistance for medical care, clothing, food, etc.)

27.Being a guardian (e.g., being the legal representative and looking after the general welfare of the person concerned)

28.Providing primary care (e.g., cleaning a wound, applying bandages)

29.Educating and training (e.g., give language courses, provide socio-professional training)

2. Table A

Wilcoxon Signed Ranked Test Results for the List of the 29 Pre-Tested Items, in French

Item	***Wilcoxon***	***p***	***Comment***
1.**Accompagner une personne dans son parcours** d'intégration, de demande d'asile, de réinsertion sociale (ex : informer la personne des procédures légales à suivre, informer la personne de la documentation nécessaire pour faire un dossier à la commune, etc.)	1702	.577	
2.**Accompagner une personne physiquement** (ex : conduire la personne à ses rdvs médicaux, l'accompagner à la commune, l'accompagner à ses formations, etc.)	4110	<.001	M_{Giving} = 4.26 **>** M_{Doing} = 2.14 *Kept as an example of volunteerism*
3.**Propager l'information en privé** (ex : discuter avec sa famille,	1046	.127	

ses amis ou collègues de la crise migratoire)			
4.**Propager l'information publiquement** (ex : partager des informations sur les réseaux sociaux, rédiger un article sur le thème, écrire dans un blog)	135	<.001	*MGiving* = 2.50 **<** M_{Doing} = 3.65 *Kept as item for Int. Acting*
5.**Travailler en tant que bénévole dans une association** (ex : nettoyer les lieux, faire l'accueil, être représentant·e, s'occuper de la logistique d'une association, etc.)	3620	<.001	M_{Giving} = 4.61 **>** M_{Doing}= 2.56 *Kept as item for Int. Giving*
6.**Faire une collecte de dons (**ex : faire du porte à porte, solliciter des personnes dans la rue, organiser un évènement caritatif, etc.)	3125	<.001	M_{Giving}= 4.37 **>** M_{Doing}= 2.83 *Kept as item for Int. Giving*
7.**Préparer des repas chauds** qui seront redistribués (ex : à des sans-abris, à des réfugié·es)	3780	<.001	M_{Giving}= 4.73 **>** M_{Doing} = 2.43 *Kept as an example of 'participating in the redistribution of…'*
8.**Faire de la promotion culturelle** (ex : organiser une exposition photo, organiser une conférence-débat, créer un projet artistique sur la situation des migrants, etc.)	659	<.001	M_{Giving} = 2.95 **<** M_{Doing} = 3.82 *Kept as item for Int. Acting*
9.**Participer à une manifestation pour montrer un soutien humanitaire** (ex : participer à une marche pour montrer du soutien aux réfugié·es dans le contexte de la crise migratoire)	1430	.714	M_{Giving} = 3.67 **<** M_{Doing} = 3.77 *Retained after discussion to be tested as Int. Acting*
10.**Participer à une manifestation pour montrer un mécontentement politique** (ex : participer à une marche pour l'ouverture des frontières dans le contexte de la crise migratoire)	435	<.001	M_{Giving} = 2.76 **<** M_{Doing} = 4.27 *Kept as item for Int. Acting*
11.**Participer à la distribution** (ex : de vêtements, de repas chauds)	4498	<.001	M_{Giving} = 4.65**>** M_{Doing} = 2.39 *Kept as item for Int. Giving*
12.**Participer au tri** (ex : de vêtements, de nourriture)	3144	<.001	M_{Giving} = 4.06**>** M_{Doing} = 2.55 *Excluded after discussion*
13.**Faire des dons** (ex : d'argent, de nourriture, de vêtements, de jouets, de couvertures, etc.)	3470	<.001	M_{Giving} = 4.45 **>** M_{Doing} = 2.54 *Kept as item for Int. Giving*
14.**Organiser un divertissement** (ex : organiser un concert, une pièce de théâtre, diffuser un film, pour divertir les personnes dans un home)	2493	.007	M_{Giving} = 3.39 **>** M_{Doing} = 2.83 *Excluded after discussion*
15.**Organiser une activité** (ex : organiser un match de foot, un atelier de dessin, ou toute autre activité participative)	2150	.002	M_{Giving}= 3.26 **>** M_{Doing}= 2.70 *Excluded after discussion*
16.**Loger/Héberger** (ex : héberger de façon temporaire un·e réfugié·e)	3053	<.001	M_{Giving} = 4.51 **>** M_{Doing} = 2.85 *Kept as item for Int. Giving*
17.**Informer les personnes concernées** (ex : informer les réfugié·es du fonctionnement d'une association, de leurs droits, du fonctionnement de la STIB)	1137	.122	
18.**Aider dans les installations** (ex : aider à installer des tentes, les toilettes, aider à réaménager un appartement qui servira de lieu d'hébergement, aider à monter un stand de soins médicaux)	2894	<.001	M_{Giving} = 4.17 **>** M_{Doing} = 2.88 *Kept as an example of volunteerism*
19.**Proposer ses expertises gratuitement** (ex : soins médicaux, aide légale, traduction, consultation psychologique, conseiller,	2993	<.001	M_{Giving} = 4.50 **>** M_{Doing} = 3.06 *Excluded after discussion*

etc.)			
20.**Inciter des personnes à se mobiliser**	969	.072	$M_{Giving} = 3.32 < M_{Doing} = 3.64$<br>*Kept as item for Int. Acting*
21.**Apporter du soutien moral et/ou psychologique** (ex : écouter les récits de vie, discuter avec des réfugié·es dans une plateforme citoyenne, discuter avec les personnes âgées dans un home, etc.)	3848	<.001	$M_{Giving} = 4.34 > M_{Doing} = 2.71$<br>*Kept as item for Int. Giving*
22.**Actions politiques** (ex : voter au niveau local ou national, faire grève)	57.5	<.001	$M_{Giving} = 1.88 < M_{Doing} = 4.60$<br>*Kept as item for Int. Acting*
23.**Signer une pétition pour montrer du soutien humanitaire**	1681	.621	
24.**Signer une pétition pour montrer un mécontentement politique**	324	<.001	$M_{Giving} = 2.60 < M_{Doing} = 4.19$<br>*Kept as item for Int. Acting*
25.**Être Parrain/Marraine** (ex : créer du lien, apporter des conseils, écouter, la personne concernée)	3492	<.001	$M_{Giving} = 4.08 > M_{Doing} = 2.33$<br>*Excluded after discussion*
26.**Être Commanditaire** (apporter de l'aide financière pour les soins médicaux, vêtements, nourriture, etc.)	2741	<.001	$M_{Giving} = 4.16 > M_{Doing} = 2.86$<br>*Excluded after discussion*
27.**Être tuteur·trice** (ex : être le/la représentant•e légal)	2231	<.001	$M_{Giving} = 3.77 > M_{Doing} = 3.00$<br>*Excluded after discussion*
28.**Donner des soins primaires** (ex : nettoyer une plaie, mettre des bandages)	3193	<.001	$M_{Giving} = 4.03 > M_{Doing} = 2.31$<br>*Excluded after discussion*
29.**Éduquer et Former** (ex : donner des cours de langues, donner une formation socioprofessionnelle)	1682	.019	*MGiving* = 3.81 > $M_{Doing} = 3.41$<br>*Excluded after discussion*

3. Table B

EFA: Factor Loadings and Communalities of the Dimensions Intergroup Giving and Intergroup Acting, with Intergroup Acting Item 1

		Factor 1	Factor 2	Uniqueness
Intergroup Giving Item 1	Working as a volunteer in an association (e.g., greeting people, cleaning the premises, taking care of logistics, etc.)	.74906	-.03450	.464
Intergroup Giving Item 2	Collecting donations (e.g., going door to door, soliciting people on the street, organizing a charity event, etc.)	.53123	.15841	.607
Intergroup Giving Item 3	Preparing hot meals to be redistributed (e.g., to the homeless, to refugees)	.88715	-.06835	.270
Intergroup Giving Item 4	Donating money, food, clothes, toys, etc.	.35954	.22953	.734
Intergroup Giving Item 5	Participating in the distribution of clothes, meals, etc.	.4050	.00686	.288
Intergroup Giving Item 6	Providing temporary accommodation/housing	.36378	.11979	.809
Intergroup Giving Item 7	Providing moral and/or psychological support (e.g., listening to life stories, talking)	.65470	.04609	.539
Intergroup Acting^ Item 1	Doing cultural promotion (e.g., organize a photo exhibition, organize a conference-debate, create an art project about migrants, etc.)	.58199	.09594	.595
Intergroup Acting Item 2	Participating in an event to show humanitarian support	-.00609	.81530	.340
Intergroup Acting Item 3	Participating in a demonstration (e.g., to show political dissatisfaction with a refusal by the authorities)	-.03645	.86277	.286
Intergroup Acting Item 4	Encouraging people to mobilize to carry out a collective action	.31315	.56953	.396
Intergroup Acting Item 5	Participating in political actions (e.g., voting at local or national level)	-.07931	.61348	.557
Intergroup Acting Item 6	Petitioning to show political discontent	-.00699	.66927	.667
Intergroup Acting Item 7	Spreading information publicly (e.g., sharing information on social networks, writing an article on the topic, writing in a blog)	.10839	.50028	.683

Note. 'Principal axis factoring' extraction method was used in combination with a 'oblimin' rotation.

Note.^ this item was excluded from the final version
